# Diversity and distribution of potential polyethylene terephthalate and polypropylene plastic-degrading bacteria communities in deep-sea cold-seep ecosystems

**DOI:** 10.1128/spectrum.01632-26

**Published:** 2026-10-07

**Authors:** Yongji Huang, Jingchun Feng, Qixuan Wu, Yingli Zhou, Si Zhang, Bingchuan Tian, Yechen Miao

**Affiliations:** 1 Guangdong Basic Research Center of Excellence for Ecological Security and Green Development, Southern Marine Science and Engineering Guangdong Laboratory (Guangzhou) 606379 , Guangzhou, China; 2 Guangdong Basic Research Center of Excellence for Ecological Security and Green Development, School of Ecology, Environment and Resources, Guangdong University of Technology 846109, Guangzhou, China; 3 South China Sea Institute of Oceanology, Chinese Academy of Sciences 74718, Guangzhou, China; 4 Higentec Co., Ltd., Changsha, China; 5 Hainan Snailfish Deepsea Technology Co., Ltd., Sanya, China; Ocean University of China, Qingdao, Shandong, China

**Keywords:** cold seep, polypropylene, polyethylene terephthalate, plastic-degrading communities

## Abstract

Biodegradation represents a promising strategy for the remediation of marine plastic pollution. Notably, deep-sea cold-seep ecosystems nurture exceptionally abundant microbial hydrocarbon-degrading bacterial resources. Due to structural similarities between plastics and natural hydrocarbons in cold seeps, cold-seep microorganisms may possess potential enzymatic strategies suited to plastic breakdown. Therefore, this study combined 1 year *in situ* inoculation and 168 days of laboratory enrichment experiments to investigate the diversity and distribution characteristics of cold-seep bacteria that respond to polyethylene terephthalate (PET) and polypropylene (PP) degradation. Enriched communities were able to grow utilizing plastics as a sole carbon source, and distinct grooves and cracks were observed on plastic films following enrichment. During cultivation, taxa that appeared unable to utilize plastics declined in abundance, while putative plastic-degrading taxa became enriched. Amplicon sequence variant (ASV) abundance dynamics revealed 24 candidate PET-degrading and 22 candidate PP-degrading ASVs, which exhibited an expanded spectrum of plastic degradation capabilities compared with literature reports. Furthermore, these putative degraders were widespread in natural cold seeps, and nutrient-rich sites appeared more conducive to the enrichment of plastic degraders. This work broadens the understanding of the plastic-degrading potential of cold-seep ecosystems and identifies candidate taxa for further biochemical and biodegradation characterization.

**IMPORTANCE:**

Plastic pollution is accumulating in the ocean, including the deep sea, where removal is extremely difficult, and natural breakdown is poorly understood. This study explores whether microorganisms from deep-sea cold-seep ecosystems can help degrade common plastics such as polyethylene terephthalate and polypropylene. Cold seeps naturally contain methane and petroleum-like hydrocarbons, so their microbes may already have biological tools for breaking down plastic-like compounds. By combining year-long deep-sea exposure with laboratory enrichment, this work identified bacterial groups that increased in abundance when plastics were the only available carbon source and observed visible damage on plastic surfaces. These findings suggest that cold seeps may act as natural reservoirs of previously overlooked plastic-degrading microbes. The study expands our understanding of how plastics may persist or break down in deep marine environments and provides candidate bacteria for future biodegradation research.

## INTRODUCTION

The extensive production and improper disposal of plastics have resulted in the annual input of approximately 9–14 million metric tons of plastic waste into the ocean (1, 2). Due to their persistence and enzyme resistance, plastics represent a long-term threat to marine organisms and can gradually accumulate along food chains, ultimately affecting top predators (3, 4). Plastic ingestion by aquatic organisms may cause digestive tract obstruction, metabolic disorders, oxidative stress, and other physiological impairments (5). Such organism-level effects may further translate into population- and community-level consequences, thereby posing broader risks to marine biodiversity and ecosystem functioning (6). Therefore, the remediation of marine plastic pollution is critical for protecting marine ecosystems and maintaining ocean health.

Microbial degradation has been regarded as an eco-friendly, cost-effective, and promising strategy for mitigating plastic contamination in marine environments (7). In general, plastic biodegradation involves four interrelated steps: (i) microbial attachment and biofilm formation on polymer surfaces; (ii) extracellular enzymatic modification or depolymerization of the polymer; (iii) uptake and intracellular metabolism of soluble oligomers or monomers; and (iv) final mineralization of oligomers to generate CO_2_, H_2_O, and CH_4_ (8, 9). To date, numerous plastic-degrading microorganisms have been isolated from soils, landfills, compost, wastewater, and coastal environments, including *Pseudomonas* sp. (10, 11), *Bacillus* sp. (12, 13), *Rhodococcus* sp. (14), and *Ideonella sakaiensis* (15). However, the degradation efficiencies of most reported strains remain relatively low, with plastic weight loss generally ranging from only 1% to 10% after 30 days to 1 year of incubation (16), and their activity under deep-sea conditions remains largely uncertain. Terrestrial and shallow coastal environments differ fundamentally from deep-sea habitats in temperature, hydrostatic pressure, salinity, oxygen availability, nutrient supply, and microbial evolutionary history. Consequently, terrestrial-derived plastic-degrading bacteria may not adequately represent the taxa or enzymatic systems that function in deep marine environments. This distinction is particularly important because the deep sea is increasingly recognized as a final sink for microplastics transported by marine snow, fecal pellets, biofouling-induced sinking, and sedimentation processes (17–19). Therefore, investigating plastic-associated bacteria directly from deep-sea environments is essential for understanding the actual ecological fate and biodegradation potential of microplastics in the ocean.

Deep-sea cold-seep ecosystems provide a particularly important yet underexplored setting for studying microbial plastic degradation. Cold seeps are chemosynthesis-based ecosystems characterized by low temperature, high hydrostatic pressure, darkness, strong redox gradients, and the continuous seepage of methane and petroleum-derived hydrocarbons (20–22). These conditions support diverse microbial communities and functional genes involved in the degradation of complex organic matter and hydrocarbons, including alkane and aromatic compound transformation pathways (23). For example, cold seep-associated *Desulfosarcina*, *Parvibaculum*, and *Erythrobacter* have been reported to participate in the oxidation of short- and long-chain alkanes through enzymes such as (1-methylalkyl)succinate synthase and alkane hydroxylase (23–25). Such metabolic capabilities are ecologically relevant to plastic degradation because polyethylene (PE) and polypropylene (PP) are hydrocarbon polymers, and polyethylene terephthalate (PET) contains aromatic terephthalate units as well as ester bonds that may be attacked by microbial hydrolases (9, 26). Therefore, cold-seep microorganisms may possess enzymatic systems that partially overlap with those involved in the degradation of natural hydrocarbons, microbial lipids, and other recalcitrant organic compounds. This potential makes cold seeps more mechanistically relevant to marine plastic biodegradation than many terrestrial or shallow coastal environments.

The Haima cold seep in the South China Sea represents an ideal natural laboratory for exploring this process. Recent evidence has shown that microplastics occur widely in seawater, sediments, and cold seep-associated organisms in the Haima cold-seep area (27, 28). The dominant polymer types include PET, PE, PP, polyamide, and polyvinyl chloride, with microplastic concentrations reaching 100 mg/kg–500 mg/kg dry sediment (27). Meanwhile, Haima cold-seep sediments harbor diverse oil- and alkane-degrading microorganisms, suggesting a strong potential for hydrocarbon transformation (23). However, despite these distinctive environmental and microbial characteristics, the diversity, enrichment patterns, and degradation potential of plastic-degrading bacteria in deep-sea cold-seep ecosystems remain poorly understood.

**Fig 1:**
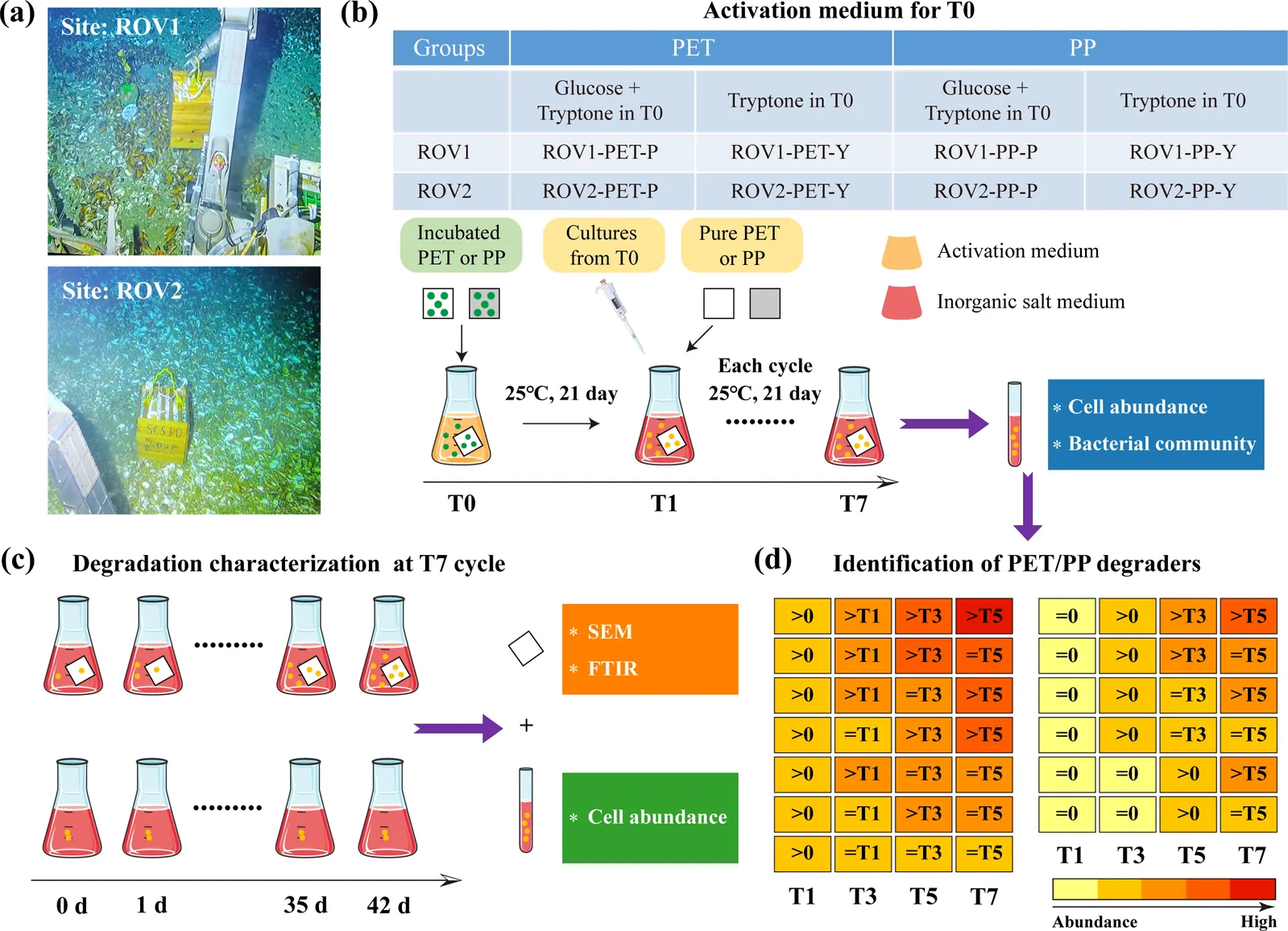
Overview of experimental workflow. (**a**) *In situ* deployment of PET and PP plastics on the seabed surface at stations ROV1 and ROV2 in the Haima cold seeps for 1 year. (**b**) Experimental enrichment of PET/PP-degrading bacteria, comprising an initial activation stage (T0) and seven enrichment cycles (T1–T7). (**c**) Characterization of PET/PP biodegradation performance after enrichment cultures at T7 cycle. (**d**) Identification of candidate PET/PP degraders based on amplicon sequence variant (ASV) abundance dynamics.

In this study, potential PET- and PP-degrading bacterial communities were enriched through the *in situ* incubation of PET and PP plastics in the Haima cold seeps for 1 year, followed by laboratory-based multi-stage enrichment experiments lasting 168 days (Fig. 1). By combining plastic degradation characterization with high-throughput sequencing of enriched bacterial communities, this study aimed to (i) evaluate the PET and PP degradation potential of enriched bacterial communities derived from cold-seep plastisphere biofilms; (ii) investigate the diversity and relative abundance of potential PET- and PP-degrading bacterial taxa in the plastisphere; (iii) and clarify the distribution patterns of these potential plastic-degrading bacteria in natural cold-seep environments. This work provides new insights into the microbial ecological mechanisms controlling PET and PP persistence in deep-sea cold-seep ecosystems and helps assess whether cold seeps may serve as reservoirs of novel marine plastic-degrading bacteria.

## MATERIALS AND METHODS

### Deployment and recycling of plastics in cold-seep areas

The experimental PET and PP films were purchased from Tiansheng Plastic Materials Co., Ltd. The films consisted of pure resin products without any additives. The thickness of each plastic film was 0.3 mm, and the plastic films were cut into sheets measuring 0.5 × 0.5 cm before use. Before *in situ* deployment, the PET and PP films were sequentially rinsed with 75% ethanol and sterile water to remove potential surface contaminants and microorganisms. Next, 5 g of each plastic film was sealed in a mesh, and three meshes containing PET or PP films were further placed in a wooden box. The *in situ* inoculation sites were located at the ROV1 and ROV2 stations surrounded by juvenile and adult mussels in the Haima cold seeps (16°43′N, 110°28′E), and the wooden boxes were placed on the surface of the seafloor in June 2022 (Fig. 1a). After 1 year of inoculation, the plastic films were retrieved. A portion of the biofilm was stored at 4°C for use as the microbial source in subsequent enrichment experiments conducted 1 month later.

### Plastic-degrading bacteria communities enrichment experiment

Two types of media were used in this study: activation medium and enrichment medium. The enrichment medium consisted of a modified inorganic salt medium (Table S1) supplemented with PET or PP films at 10 g/L as the sole carbon source. Because microbial biomass and activity on the *in situ* plastic films were expected to be low, an initial activation step was performed before enrichment. The activation medium was prepared using the same inorganic salt medium supplemented with either 0.01% tryptone, designated medium Y, or 0.01% tryptone plus 1% glucose, designated medium P (Fig. 1b), following the reported method (29). The pH of all media was adjusted to 7.0, and the media were sterilized at 121°C for 20 min.

Plastic-associated microbial communities were enriched according to plastic type. For each sampling site (ROV1 and ROV2), three independent PET- or PP-associated plastic film samples were collected and used separately as inoculum sources, resulting in three biological replicates for each site-plastic type combination. Specifically, *in situ* PET- or PP-associated plastic films were inoculated into 20 mL of activation medium and incubated for 21 days as the initial activation stage, designated cycle T0. After activation, 2 mL of cultures were transferred into 20 mL of enrichment medium to initiate the first enrichment cycle, designated T1. Subsequent enrichment cycles were performed in the same manner by transferring 2 mL of enrichment culture into fresh enrichment medium at the end of each 21 day cycle. In total, seven enrichment cycles were performed and designated T1 to T7. All cultures were incubated at 25°C with shaking at 120 rpm for 21 days per cycle. Although these incubation conditions differ from the low-temperature and high-pressure conditions typical of cold-seep environments, they were selected to improve enrichment efficiency and to verify whether the cold-seep plastic-associated microbiome harbored microorganisms with potential plastic-degrading capacity. Therefore, the enriched communities should be interpreted as laboratory-selected assemblages, potentially biased toward mesophilic and piezotolerant taxa, rather than as direct representatives of *in situ* plastic degradation activity. Microbial growth at the end of each cycle was monitored by measuring optical density at 600 nm (OD_600_). Genomic DNA was extracted from enrichment cultures collected on day 21 of cycles T1, T3, T5, and T7 for subsequent microbial community analysis. A schematic overview of the experimental workflow is depicted in Fig. 1b.

### Characterization of plastic degradation performances

Cycles T1–T6 were conducted as regular 21 day enrichment cycles. To better characterize the degradation properties of the plastics, cultures were sampled on day 21 for DNA extraction in the T7 cycle as in the previous cycles, but the incubation was further extended to 42 days for degradation characterization. During this extended T7 stage, bacterial abundance in the culture medium was determined by hemocytometer counting under a light microscope on days 0, 1, 2, 4, 7, 10, 14, 21, 28, 35, and 42, and the PET and PP films collected on day 42 were used for subsequent Fourier transform infrared (FTIR) spectroscopy and scanning electron microscopy (SEM) degradation-performance analyses (Fig. 1c). Blank control treatments were prepared by adding PET or PP films to uninoculated medium under the same conditions as the inoculated cultures. The inoculated and control plastic samples for degradation characterization were processed with the following procedures. Plastics with attached microbes were fixed for 3 h using 2.5% glutaraldehyde in 0.1 mM phosphate buffer containing 2.8% NaCl. The fixed plastic samples were washed in ultrapure water and dehydrated via gradient ethanol (30, 50, 70, and 95%) for 5 min. Finally, the plastics were lyophilized in a vacuum freeze dryer. To characterize changes in surface morphology and functional groups, the plastics were sequentially washed with ultrapure water containing 1% SDS, ultrapure water, and 75% ethanol in an ultrasonic cleaning bath for 10 min to remove bacteria and debris. The cleaned plastic samples were air-dried at 25°C. The surface morphology of initial plastics, bacteria-colonized plastics, and clean plastics was observed using an SEM (Hitachi, SU8220, Japan) equipped with an energy-dispersive X-ray spectrometer. The functional groups of initial plastics and clean plastics were analyzed using FTIR spectroscopy (Thermo Nicolet Co., NEXUS 670, USA), and the scanning area ranged from 500 cm^−1^ to 4,000 cm^−1^.

### Characterization of bacterial communities

Genomic DNA was extracted from 8 mL of enrichment culture using the PureLink Genomic DNA Kit (Invitrogen, Carlsbad, CA, USA) following the manufacturer’s protocol. Amplicon libraries targeting the V3–V4 hypervariable region of the 16S rRNA gene were prepared using primer pair 341F (5′-CCT ACG GGN GGC WGC AG-3′) and 806R (5′-GGA CTA CHV GGG TWT CTA AT-3′) (30), each of which carried a unique 6-bp barcode at the 5′ end to enable sample demultiplexing. Polymerase chain reaction (PCR) amplifications were performed under the following cycling conditions: initial denaturation at 94°C for 5 min; 30 cycles of 94°C for 15 s, 53°C for 15 s, and 72°C for 30 s; with a final extension at 72°C for 5 min. The PCR products were purified using DNA Clean Beads (Vazyme, N411-01, China) and quantified on a Qubit fluorometer (Thermo Fisher Scientific, Waltham, MA, USA). Equimolar amounts of each PCR product were pooled to construct sequencing libraries, which were then sequenced on the Illumina NovaSeq-PE250 platform (Novogene Co., Ltd., USA).

Raw paired-end reads were processed in Quantitative Insights Into Microbial Ecology (QIIME 2, version 2025.7) (31). Sequences were demultiplexed using sample-specific barcodes, and primers were trimmed using the cutadapt plugin. The reads were subsequently quality-filtered, denoised, merged, and checked for chimeras with the DADA2 (32) plugin to generate amplicon sequence variants (ASVs) with 100% similarity and obtain a feature table and representative sequences. The taxonomic assignment of individual ASVs was performed using the feature-classifier plugin against the SILVA 138.2 reference database.

### Identification of putative PET/PP-degrading bacteria

Microbial diversity typically declines when a community is challenged with a single carbon source in enrichment culture (33). If a bacterial group can grow independently and maintain a consistently increasing relative abundance when cultured under a single plastic carbon source, it can be inferred that the bacterial species may possess the ability to degrade and metabolize plastic as a carbon and energy source. According to this principle and the reported identification method (29), this study identified putative PET- and PP-degrading bacteria in the enriched microbial communities at the ASV level. ASVs that were present in all sequencing cycles with stable or increasing relative abundance, as well as those that were below the detection limit during the early sequencing cycles but appeared later and maintained stable or increasing relative abundances, were defined as putative PET- and PP-degrading bacteria (Fig. 1d).

### Microbial community diversity analysis

For downstream diversity analysis, the feature table was rarefied to an even sequencing depth using the “vegan” package in R to control for differences in sampling effort. α-Diversity metrics of bacterial communities in PET- and PP-enriched cultures were calculated in the “vegan” package and reported as the mean ± standard deviation, including observed ASVs, Chao1 richness, Faith’s phylogenetic diversity, Shannon diversity, and Good’s coverage. Temporal shifts in the relative abundances of bacterial genera over successive enrichment cycles were visualized as stacked bar charts. Based on the ASVs identified for putative PET- and PP-degrading bacteria, the ASV sequences of the degrading bacteria were designated and screened from the representative sequences. The representative sequences were aligned using MUSCLE, and a maximum-likelihood phylogenetic tree was constructed using IQ-TREE under the K2P+G4 substitution model. Branch support was assessed using 1,000 ultrafast bootstrap replicates and 1,000 SH-aLRT replicates. The resulting tree was visualized and annotated using the Interactive Tree of Life platform (http://itol.embl.de/).

### Environmental geochemical measurements

To assess the environmental distribution of potential plastic-degrading bacteria in natural cold-seep environments, seawater and sediment samples from five cold-seep sites were analyzed for geochemical parameters. These five sites were located in the Haima cold seep (16°43′N, 110°28′E), with a sampling depth of approximately 1,400 m. ROV1 was colonized by juvenile mussels, ROV2 was characterized by abundant adult mussel communities, ROV3 was associated with clams, ROV4 was mainly colonized by sea anemones, and ROV5 was also characterized by clam communities. Methane (CH_4_) concentrations were analyzed using a gas chromatograph (TRACE 1300, Thermo Fisher Scientific, Waltham, MA, USA). Total organic carbon (TOC) and total inorganic carbon were determined by a TOC-L analyzer (Shimadzu, Kyoto, Japan). Inorganic nutrients (NO_3_^−^, NO_2_^−^, NH_4_^+^, and PO_4_^3−^) were measured using an automated nutrient analyzer (Lachat QuikChem 8500 S2, Hach, Loveland, CO, USA). Sulfate (SO_4_^2−^) content was determined using an ion chromatograph (Aquion, Thermo Fisher Scientific, USA). Divalent cations (Ca^2+^ and Mg^2+^) were quantified via inductively coupled plasma optical emission spectrometry (iCAP 7000 Series, Thermo Fisher, Waltham, MA, USA).

### Statistical analysis

The abundances of the above-identified PET- and PP-degrading ASVs in cold-seep seawater and sediment samples from five cold-seep sites were visualized using bar charts and bubble charts. Mantel tests were performed using the “LinkET” package to identify a subset of key environmental variables explaining variability in plastic-degrader abundance. Canonical correspondence analysis (CCA) was applied to detect correlations between environmental geochemical parameters and the abundance of plastic-degrading bacteria. Prior to CCA, the environmental factors were screened for multicollinearity, and factors with a variance inflation factor >10 were excluded.

## RESULTS AND DISCUSSION

### Long-term enrichment of plastic-degrading bacteria communities

**Fig 2:**
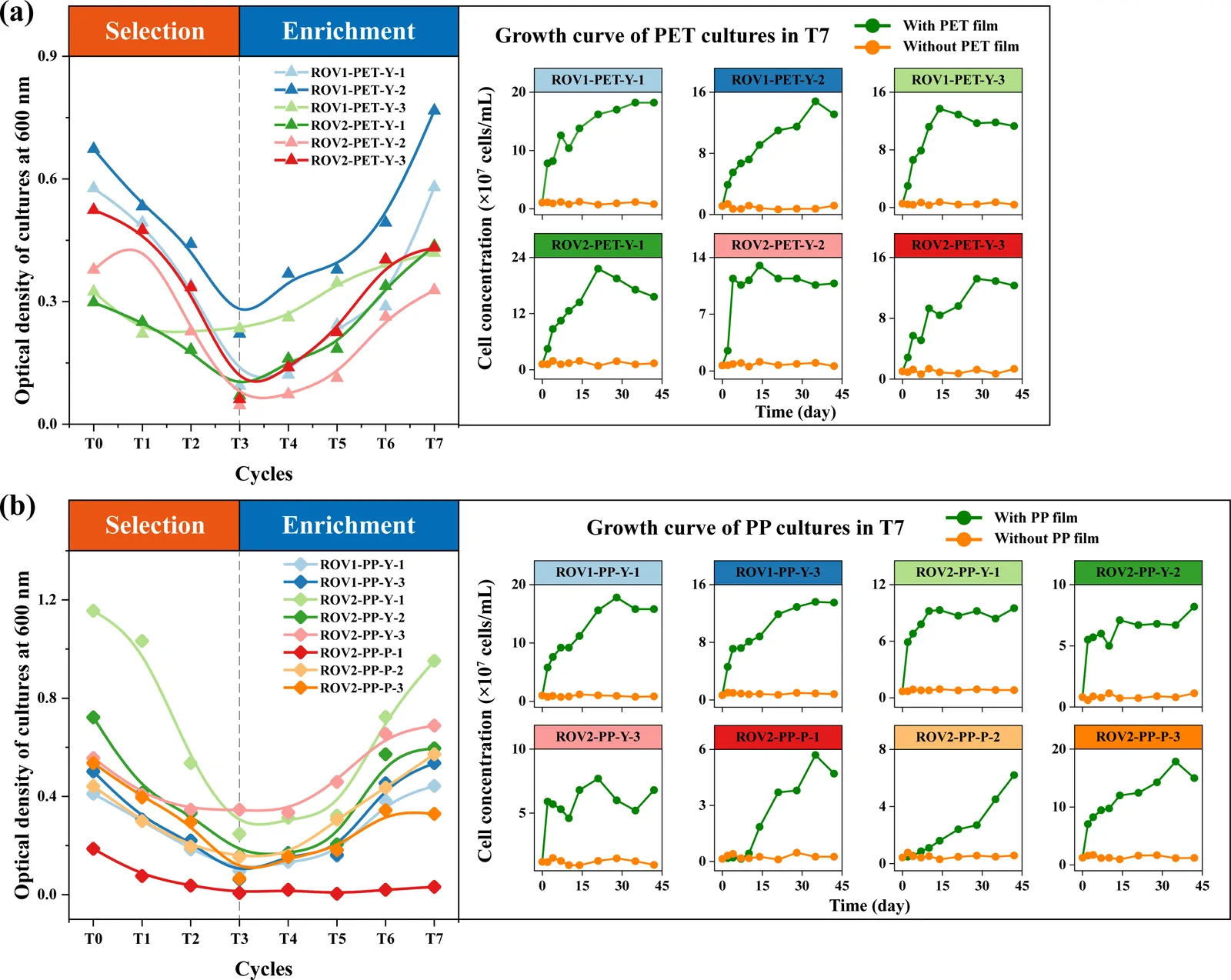
Bacterial abundance curve of PET and PP enrichment cultures. (**a**) Optical density at 600 nm of PET enrichment cultures at the end of the incubation cycle from stages T0 through T7, and growth curves of PET enrichment cultures over 42 days during the T7 cycle. (**b**) Optical density at 600 nm of PP enrichment cultures at the end of the incubation cycle from stages T0 through T7, and growth curves of PP enrichment cultures over 42 days during the T7 cycle.

In this study, one activation cycle followed by seven enrichment cycles was conducted to enrich plastic-degrading bacteria on *in situ* PET and PP plastic films collected from the Haima cold seeps. By the conclusion of the experiment, measurable bacterial activity was retained in only 14 of the 24 enrichment cultures, while the remaining 10 cultures were rendered nonviable over successive cycles. Figure 2 shows the OD_600_ of the enrichment cultures at the end of each cycle. Except for ROV1-PET-Y-3 and ROV2-PP-P-1, a decline in OD_600_ was observed before the T3 cycle, after which a steady increase was recorded through the T7 cycle. For example, the OD_600_ of the ROV1-PET-Y-1 culture decreased from 0.577 at T0 to 0.094 at T3, and then gradually rebounded to 0.580 by T7. These consistent trends indicated that the T3 cycle marked a transition point for the enrichment culture of plastic-degrading bacteria. During cycles T0–T3, non-degrading strains were progressively eliminated, and plastic-degrading bacteria were selectively enriched from cycle T3 onward, leading to a marked elevation in cell abundance. Consequently, the period from T0 to T3 was designated the selection stage, while the period from T3 to T7 was denoted as the enrichment stage. This community abundance enrichment dynamic was consistent with classical selection mechanisms under matrix-specific enrichment conditions (34, 35). During the initial selection stage, many bacterial taxa incapable of utilizing plastic polymers persisted. When soluble carbon became limited and enrichment systems underwent multiple passages, these non-plastic-degrading taxa were progressively eliminated and diluted, leading to a decline in overall bacterial abundance (36). Over successive passages, taxa capable of colonizing plastic surfaces, secreting depolymerizing extracellular enzymes, and utilizing plastic degradation products were selectively enriched. Consequently, these functional taxa were gradually amplified and ultimately dominated the enrichment cultures. The abundance of these functional taxa expanded, driving the biomass increase observed after the T3 cycle. Similar succession patterns and the emergence of specialized plastic-associated communities have been reported in previous continuous enrichment studies and plastisphere surveys on environmental plastic debris (34, 37, 38).

The effects of different activation media formulations on bacterial enrichment were also evaluated. When 0.01% tryptone and 1% glucose were employed as the activated carbon source, PET-degrading bacteria were not enriched. In the PP cultures, only ROV2-PP cultures supplemented with glucose maintained growth, yet extremely low OD_600_ values were recorded for ROV2-PP-P-1. However, when 0.01% tryptone was used, only one of the enriched PP cultures failed to grow (ROV1-PP-Y-2). These findings imply that the addition of glucose as an activating carbon source may impede the enrichment and subsequent isolation of PET- and PP-degrading bacteria from cold-seep environments. The inhibitory effect of adding readily available glucose during the activation phase provided crucial experimental support for the selection stage. Readily available soluble carbon sources relieve selection pressure for the energetically costly production of extracellular depolymerases, resulting in the downregulation of enzymes required to degrade structurally complex substrates (39). Consequently, in the present study, non-degradative taxa that exploited the simple carbon source expanded during the activation stage and outcompeted populations producing degradation enzymes. This dynamic reduced the probability that plastic-degrading taxa would be enriched or isolated in enrichment cultures supplemented with glucose.

The impact of supplying PET or PP films as the sole carbon source on the growth dynamics of enriched cultures was evaluated during the T7 enrichment cycle (Fig. 2). When cultures from the previous enrichment cycle were transferred into inorganic salt medium lacking PET or PP films, microbial growth was severely constrained. The cell abundances in all control groups remained essentially constant or exhibited a slight decline over the 42 day incubation period. By contrast, inoculation into media containing PET or PP films produced rapid exponential growth, with a significant increase in bacterial numbers within the first 14 days. After the initial phase, the growth trajectories of the cultures diverged. Bacterial abundance continued to increase slowly in some cultures, including ROV1-PET-Y-1. In others, a stationary phase was reached, consistent with the limitation of accessible nutrients and space. These observations indicated that the enriched microbial microbiota were able to degrade PET and PP and utilize the resulting carbon compounds to sustain growth and reproduction.

In this study, microbial communities enriched from deep-sea cold-seep plastispheres maintained growth with PET or PP as the sole carbon source and showed evidence of plastic transformation under laboratory enrichment conditions. Together with our previous detection of microplastic contamination in cold-seep seawater and sediments, these findings suggest that cold-seep plastispheres may serve as potential reservoirs of candidate plastic-degrading microorganisms and provide a useful system for investigating plastic-carbon cycling in deep marine ecosystems.

### Plastic degradation properties of enriched microbial communities

**Fig 3:**
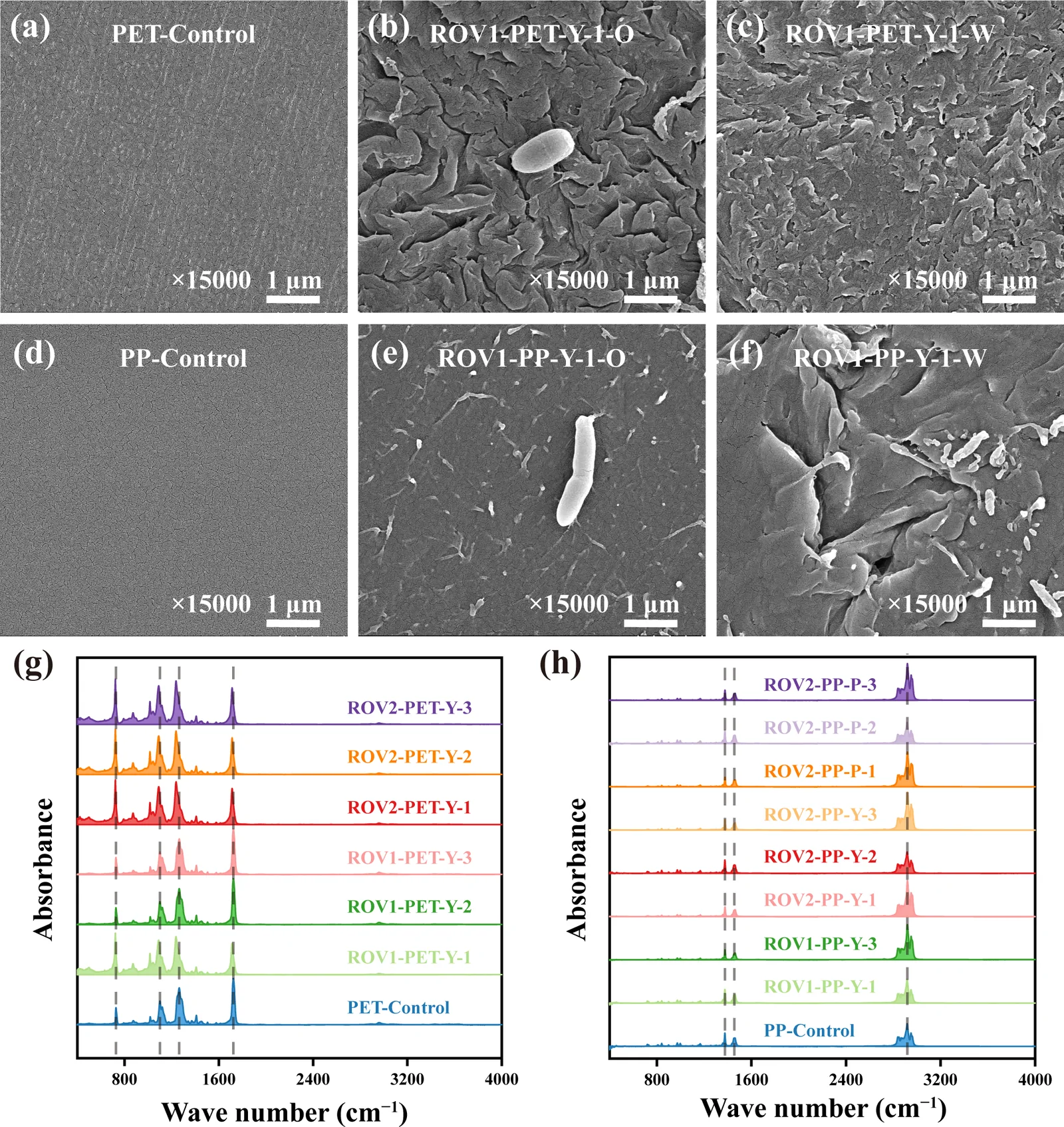
Biodegradation performance characterization of PET and PP films after enrichment cultures on the 42nd day of the T7 cycle. Scanning electron micrographs of (**a**) control PET film, (**b**) fixed PET film, (**c**) washed PET film, (**d**) control PP film, (**e**) fixed PP film, and (**f**) washed PP film after the enrichment experiment, revealing changes in the surface morphology of plastic films under enrichment cultures. FTIR spectra of (**g**) PET film and (**h**) PP film illustrating the changes in functional groups on plastic films under enrichment cultures.

Bacterial growth dynamics differed between the enrichment cultures (Fig. 2). In the ROV1-derived cultures, ROV1-PET-Y-2 showed a marked decrease in bacterial abundance on day 42. In the ROV2-derived cultures, ROV2-PET-Y-1 began to decline after day 21, whereas ROV2-PP-P-1 and ROV2-PP-P-3 showed a marked decrease on day 42. Accordingly, the enrichment experiment was terminated on day 42 of the T7 cycle, and PET and PP film samples were collected for the subsequent characterization of plastic-degrading activity. SEM observations were performed to examine the surface morphology of PET and PP films from the ROV1-PET-Y-1 and ROV1-PP-Y-1 enriched groups, with films incubated in sterile inorganic salt medium for 42 days serving as the control (Fig. 3). In the control groups, the surfaces of PET and PP remained smooth and featureless (Fig. 3a and d). In contrast, plastic membranes from the enriched groups displayed bacterial colonization and surface-associated matrix-like materials on the polymer surfaces (Fig. 3b and e). After thorough washing to remove microbial biofilm, significant signs of biodegradation were observed on the polymer surface (Fig. 3c and f). Specifically, PET membranes exhibited newly formed groove-like features, while PP membranes displayed clear cracked and fissured structures. These observations demonstrated that the enriched microflora were able to adhere to and actively modify the surfaces of both PET and PP films.

FTIR spectroscopy was employed to examine changes in the surface functional groups of plastics following biodegradation treatment (Fig. 3g and h). PET observations revealed the maintenance of the original absorption peaks or the appearance of entirely new peaks, indicating that no gross new functional groups were produced during biodegradation. However, several PET absorption bands shifted to lower wavenumbers in the spectra of ROV1-PET-Y-1, ROV2-PET-Y-1, ROV2-PET-Y-2, and ROV2-PET-Y-3, implying the decreased vibrational frequencies of the corresponding bonds. Using ROV1-PET-Y-1 as an example, the C = O stretching vibration of ester moved from 1,724 cm^−1^ in the control to 1,713 cm^−1^ in the degradable PET. Additionally, the ester C–O stretching band shifted from 1,264 cm^−1^ to 1,236 cm^−1^, and the symmetric C–O–C/C–O stretching band of ester or ethylene glycol changed from 1,101 cm^–1^ to 1,090 cm^–1^. This weakening of vibrational frequencies in the functional groups was attributed to the hydrolysis of ester bonds mediated by extracellular enzymes secreted from enriched microbial communities. However, the spectra of ROV1-PET-Y-2, ROV1-PET-Y-3, and all PP degradation treatments displayed no appreciable shifts in the principal functional-group absorption peaks. This may reflect a low degree of chemical modification and limited biodegradation efficiency under the present experimental conditions. Therefore, the actual degradation capacity of these enrichment cultures, particularly for PP, should be interpreted cautiously and requires further validation using more sensitive quantitative methods. Taken together, SEM and FTIR analyses confirmed that the enriched microflora successfully colonized both PET and PP membranes and induced distinct surface deterioration patterns.

Microorganisms that colonize plastic surfaces establish a distinct ecological niche commonly referred to as the plastisphere (40). Plastics serve as a physical substrate for microbial attachment and biofilm formation, as well as a potential carbon source to support microbial metabolism. During the biodegradation of PET, PET-degrading microorganisms can secrete extracellular hydrolases that catalyze the hydrolysis of ester bonds, including PET hydrolases, esterases, and chitinases. These reactions generate the intermediate products mono(2-hydroxyethyl) terephthalate (MHET) and bis(2-hydroxyethyl) terephthalate, which are further hydrolyzed to generate terephthalic acid and ethylene glycol for microbial growth (41). Notably, PET hydrolases isolated from *Saccharomonospora viridis*, *Thermobifida fusca*, and *Ideonella sakaiensis* have been demonstrated to depolymerize PET and produce intermediate products such as MHET (42). By contrast, the biodegradation of PP is more challenging, principally because PP is constructed on a nonpolar and chemically inert C–C backbone and exhibits pronounced hydrophobicity. These attributes preclude the presence of readily hydrolysable functional groups. Thus, the microbial degradation of PP typically necessitates the secretion of oxidases, such as laccases and peroxidases, which introduce carbonyl and other oxygen-containing functional groups into the polymer chain through preliminary oxidation to enhance its polarity, thereby promoting subsequent microbial degradation. For example, in previous research, the analysis of PP degradation products following treatment with *Lysinibacillus* sp. JJY0216 revealed the presence of multiple aliphatic compounds containing CH_2_ units (43), which was consistent with oxidative chain breakage and the conversion of PP fragments. In summary, because PET molecules contain ester bonds that are susceptible to enzymatic hydrolysis, whereas PP lacks analogous reactive sites, PET was more readily biodegraded than PP under enrichment culture conditions (Fig. 3). It is worth noting that despite these, results indicate that the cold-seep plastic-associated microbiome harbors microorganisms with potential plastic-degrading capacity under laboratory enrichment conditions. However, because the enrichment was conducted at 25°C and atmospheric pressure, the enriched communities should be interpreted as laboratory-selected assemblages rather than direct representatives of *in situ* plastic degradation activity in deep-sea cold-seep environments.

### Temporal shifts in bacterial community composition during enrichment

The diversity of enrichment communities in the T1, T3, T5, and T7 cycles was systematically assessed. The Good’s coverage for all bacterial communities exceeded 0.999, indicating that the sequencing depth was sufficient to capture the vast majority of taxa in each sample (Fig. S1). The α-diversity of bacterial communities declined progressively over the enrichment process, with a modest rebound in the T7 cycle (Fig. S2), including observed ASVs, Chao1 richness, Faith’s phylogenetic diversity, and Shannon diversity. The changes in bacterial α-diversity may be attributed to the variability in the ability of microorganisms to utilize carbon sources. Fast-acting carbon sources such as glucose can be directly utilized by microorganisms, whereas late-acting carbon sources require microorganisms to undergo a period of adaptation before metabolism (44). In this study, because the plastics were difficult to degrade, the enriched community relied on substrates with chemically resistant (late-acting carbon) rather than easily absorbable carbon. Under this directed selection, taxa unable to metabolize polymers or their degradation products gradually disappeared during the enrichment process (33). The diversity increased slightly during the T7 cycle, possibly due to the recovery of some plastic-degrading bacteria, whose abundance was sufficient to be detected by sequencing. Previous reports have described similar patterns of α-diversity dynamics in enrichment cultures targeting bacteria degrading PE and PP plastic films in urban rivers (29).

**Fig 4:**
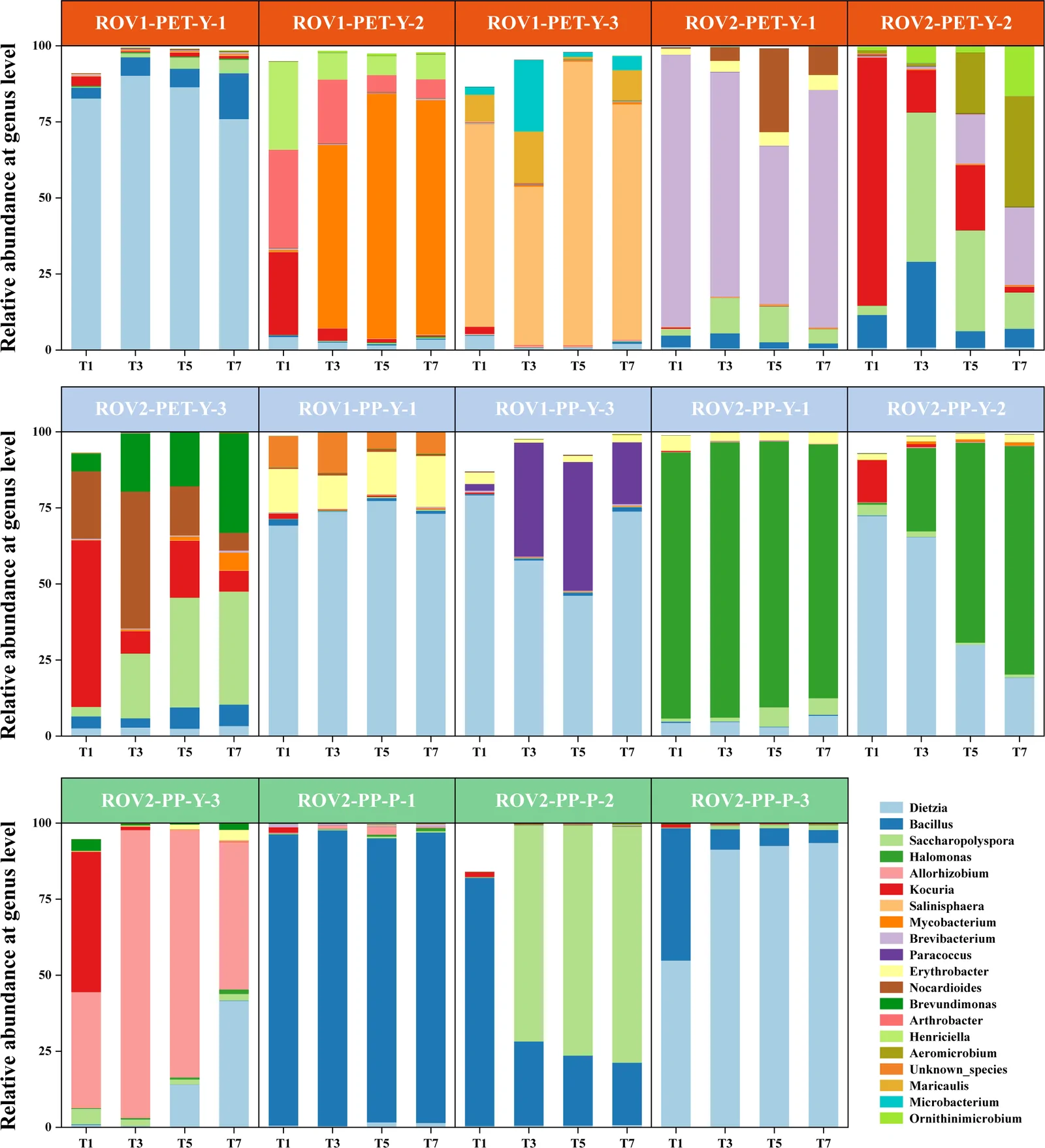
Changes in the relative abundances of different genera in the bacterial communities of enrichment cultures at the end of the incubation cycle from stages T1 through T7.

Changes in the relative abundances of different genera in enriched microbial communities were further analyzed across culture cycles, revealing three general trends (Fig. 4). These consisted of (i) taxa with high and stable relative abundances across cycles, such as *Bacillus* in the ROV2-PP-P-1 enrichment culture, which remained dominant (> 90%) throughout all cycles; (ii) taxa whose relative abundances declined over successive cycles, such as *Kocuria* in the ROV1-PET-Y-2 enrichment culture, which decreased from 27.20% at T1 to 0.39% at T7; and (iii) taxa that became increasingly dominant, including *Halomonas* in the ROV2-PP-Y-2 enrichment culture, which rose from 0.67% at T1 to 75.08% at T7. These observations indicated that bacterial taxa differed in terms of their capacities and ecological adaptations to utilize plastic or degradation intermediates when plastics were provided as the sole carbon source. The taxa that maintained high abundances across successive passages likely directly metabolized plastic-derived carbon, possessed faster growth rates, or displayed greater stress tolerance, which enabled them to outcompete other community members (45). By contrast, taxa whose abundances declined may have lacked key degradation enzymes or have been displaced by more efficient degraders (46). Taxa that rose to dominance during serial passages probably gained a competitive advantage through adaptive evolution, horizontal gene transfer, or the metabolism of primary plastic degradation products (47). Collectively, these patterns were consistent with progressive selection and the enrichment of bacteria capable of exploiting plastic-derived carbon, accompanied by a concomitant loss of taxa lacking that capability.

Additionally, although all enrichment cultures used PET or PP as the sole carbon source and underwent similar laboratory conditions, their community evolution trajectories and dominant taxa were not consistent among different enrichment treatments or even among biological replicates. The divergent community succession patterns among biological replicates may be explained by the following three reasons. Firstly, the microbial biomass on deep-sea *in situ* plastic films was relatively low, and biofilm distribution on plastic surfaces was spatially heterogeneous. Therefore, small differences in the initial inoculated plastisphere may have been amplified during the initial activation stage and subsequent enrichment cycles. Secondly, continuous transfer under selective conditions with plastic as the sole carbon source may have introduced stochastic effects. Some low-abundance microorganisms may have been lost during transfer in one replicate but successfully transferred and subsequently enriched in another replicate, resulting in divergent community trajectories. In addition, plastic-associated microbial communities may contain functionally redundant taxa that adopt different ecological strategies, including direct transformation of polymer, utilization of released oligomers or additives, secondary consumption of metabolites produced by primary degraders, biofilm formation, and opportunistic growth under carbon-limited conditions. Therefore, the differences among biological replicates may reflect microscale heterogeneity in the original plastisphere communities and multiple possible succession pathways during enrichment.

### Identification of potential PET- and PP-degrading bacteria

**Fig 5:**
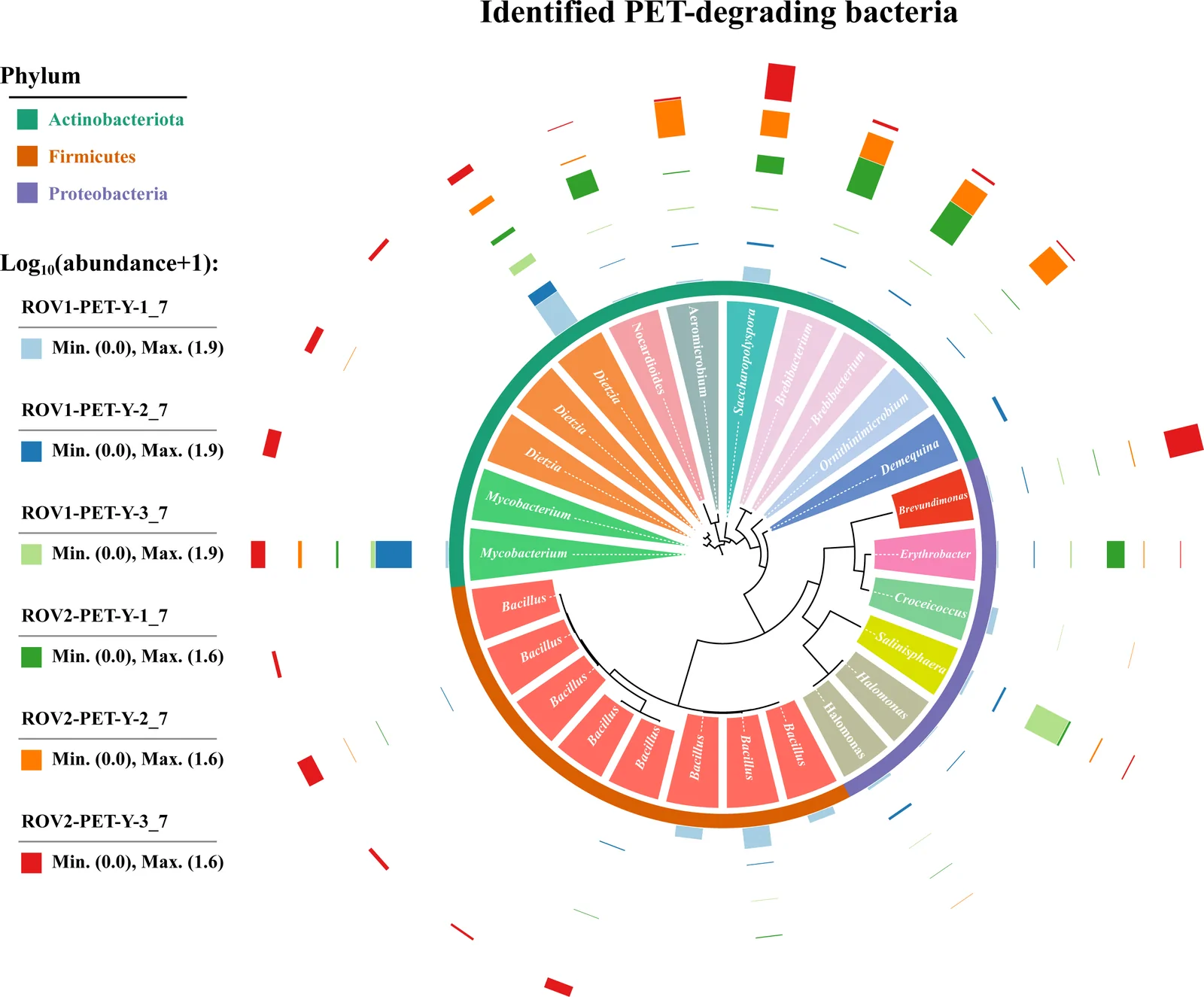
Phylogenetic tree of identified PET-degrading bacteria and their abundances in PET enrichment cultures at the T7 cycle. Representative sequences were aligned using MUSCLE, and a maximum-likelihood phylogenetic tree was constructed using IQ-TREE under the K2P+G4 substitution model. Branch support was assessed using 1,000 ultrafast bootstrap replicates and 1,000 SH-aLRT replicates. The inner colored ring indicates the phylum-level taxonomic affiliation of ASVs. The outer bar plots represent the log10-transformed relative abundance of each ASV in PET enrichment cultures at T7, with different colors corresponding to different enrichment cultures.

**Fig 6:**
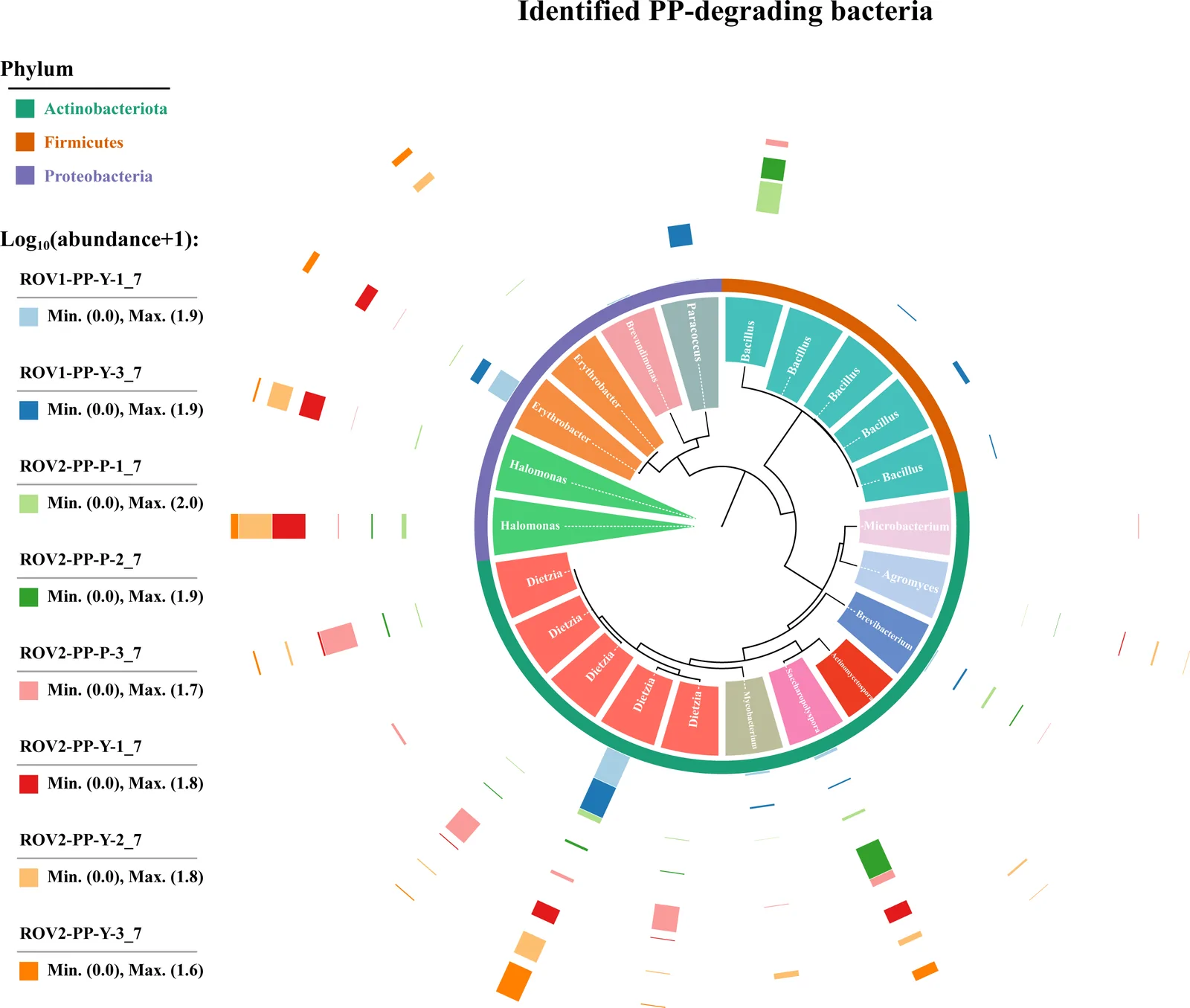
Phylogenetic tree of identified PP-degrading bacteria and their abundances in PP enrichment cultures at the T7 cycle. Representative sequences were aligned using MUSCLE, and a maximum-likelihood phylogenetic tree was constructed using IQ-TREE under the K2P+G4 substitution model. Branch support was assessed using 1,000 ultrafast bootstrap replicates and 1,000 SH-aLRT replicates. The inner colored ring indicates the phylum-level taxonomic affiliation of ASVs. The outer bar plots represent the log10-transformed relative abundance of each ASV in PP enrichment cultures at T7, with different colors corresponding to different enrichment cultures.

To date, most enrichment and isolation studies on plastic-degrading bacteria have identified dominant taxa based on species abundance, which may overlook some low-abundance but functionally important plastic degraders (48, 49). To address this limitation, this study employed an abundance-change method to identify PET- and PP-degrading bacteria in enriched communities by tracking dynamic shifts in ASV abundance across incubation cycles (Fig. 1d). Using this approach, we detected taxa whose relative abundances gradually increased or remained constant during enrichment and which therefore represent candidate plastic degraders. The phylogenetic tree and abundance distribution of PET-associated ASVs identified during cycle T7 based on abundance changes are shown in Fig. 5. A total of 26 ASVs putatively involved in PET degradation were detected across six PET-enriched cultures. These ASVs were affiliated with members of the phyla Actinobacteriota, Firmicutes, and Proteobacteria. At the genus level, these 26 ASVs were assigned to *Dietzia*, *Saccharopolyspora*, *Halomonas*, *Salinisphaera*, *Mycobacterium*, *Brevibacterium*, *Erythrobacter*, *Brevundimonas*, *Aeromicrobium*, *Nocardioides*, *Ornithinimicrobium*, *Bacillus*, *Croceicoccus*, and *Demequina*. Among these genera, *Bacillus* was represented by the largest number of ASVs (eight). Figure 6 depicts the phylogenetic tree and abundance distribution of PP-associated ASVs during the T7 cycle. From eight PP-enriched cultures, we identified 22 ASVs associated with putative PP degradation, again spanning Actinobacteriota, Firmicutes, and Proteobacteria. These 22 ASVs were assigned to *Dietzia*, *Bacillus*, *Saccharopolyspora*, *Halomonas*, *Mycobacterium*, *Brevibacterium*, *Paracoccus*, *Erythrobacter*, *Brevundimonas*, *Microbacterium*, *Agromyces*, and *Actinomycetospora*. Among these genera, *Dietzia* and *Bacillus* were the most strongly represented genera in the PP enrichment cultures at five ASVs each.

Within individual enrichment cultures, particular ASVs reached very high relative abundances. For example, ASVs affiliated with *Dietzia*, *Mycobacterium*, and *Salinisphaera* reached relative abundances of 75.91, 77.03, and 77.38%, respectively, in the ROV1-PET enrichment. In the ROV2-PET enrichment, *Brevibacterium* attained a relative abundance of 78.10%, and ASVs affiliated with *Aeromicrobium*, *Saccharopolyspora*, and *Brevundimonas* reached relative abundances of 36.23, 37.17, and 32.67%, respectively, in their respective samples. In the PP enrichments, the dominant degraders (relative abundance >30%) primarily consisted of ASVs belonging to *Dietzia*, *Bacillus*, *Saccharopolyspora*, and *Halomonas*. Importantly, each enrichment also contained a diversity of low-abundance ASVs that exhibited abundance shifts consistent with the abundance variation method, such as rare ASVs of *Brevundimonas*, *Microbacterium*, and *Agromyces*. Therefore, these results demonstrate that multiple taxa can proliferate when plastic is supplied as the sole carbon source, implying that genes and metabolic pathways involved in PET and PP degradation may be distributed across multiple species within enriched communities rather than being confined to numerically dominant taxa.

The PET- and PP-degrading bacteria identified in this study were compared with those previously reported in the literature (Table 1). Overall, the taxa recovered here exhibited a broader putative plastic-degrading spectrum. For example, the potential PET-degrading bacteria *Aeromicrobium* and *Salinisphaera* identified in this study have not yet been reported as plastic degraders. In addition, *Dietzia* and *Saccharopolyspora* displayed potential for PP degradation in addition to their previously reported PET-degrading capability. It is noteworthy that some of the low-abundance degrading bacteria identified in the present study have not been reported to exhibit plastic-degrading activity, including *Aeromicrobium*, *Croceicoccus*, and *Demequina*. In summary, utilizing an abundance-change approach allowed us to identify both dominant and low-abundance candidate PET- and PP-degrading taxa that would likely be overlooked using the dominant-abundance method alone. Nevertheless, it should be emphasized that assigning plastic-degrading capability based solely on enrichment dynamics remains preliminary. Although the enrichment of specific ASVs suggests their potential association with PET/PP transformation, ASV abundance changes alone do not establish direct plastic-degrading function. These taxa may include direct degraders, secondary consumers, commensal microorganisms, or biofilm-associated bacteria. Therefore, the functional roles of these candidate taxa require further confirmation using strain-level degradation assays and quantitative chemical analysis. Notably, several pure strains have been obtained from the enrichment cultures, and their PET- and PP-degrading activities were subsequently experimentally validated under controlled laboratory conditions, with the majority of the verified degraders belonging to the candidate taxa identified above (Table S2).

**TABLE 1:** Comparative analysis of PET- and PP-degrading bacteria identified in this study and reported in the literature

Present study	Published reports
*Aeromicrobium* (PET)	Not reported
*Actinomycetospora* (PP)	Not reported
*Agromyces* (PP)	PA (50)
*Bacillus* (PET, PP)	PE (51), PHB (52), PET (53), PHA (54), PC (55)
*Brevibacterium* (PET, PP)	PS (56)
*Brevundimonas* (PET, PP)	PS (57), PCL (58), PA (59)
*Croceicoccus* (PET)	Not reported
*Demequina* (PET)	Not reported
*Dietzia* (PET, PP)	PET (60)
*Erythrobacter* (PET, PP)	Not reported
*Halomonas* (PET, PP)	PE (61), PP (61), PET (61)
*Mycobacterium* (PET, PP)	PHA (62)
*Nocardioides* (PET)	Not reported
*Ornithinimicrobium* (PET)	Not reported
*Paracoccus* (PP)	PE (63), PHA (64), PHB (64), PHBV (64), PET (65)
*Saccharopolyspora* (PET, PP)	PET (66)
*Salinisphaera* (PET)	Not reported

### Distribution of identified PET- and PP-degrading bacteria in cold-seep environments and key environmental drivers

**Fig 7:**
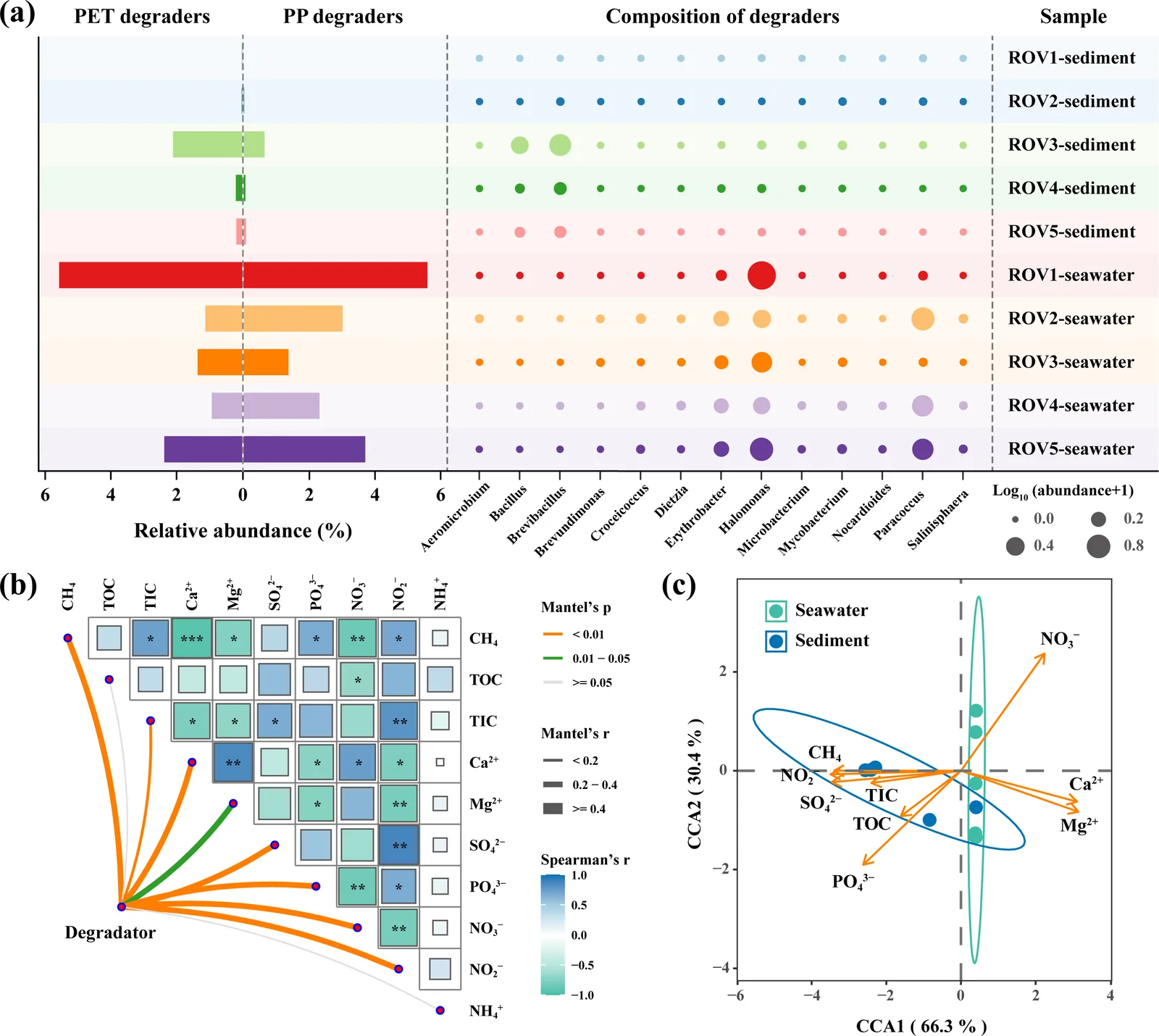
Distribution characterization of identified PET- and PP-degrading bacteria in cold-seep environments and key environmental drivers. (**a**) Abundances of identified plastic-degrading bacteria in natural cold-seep seawater and sediment communities. (**b**) Mantel correlation analysis identifying a subset of key environmental variables explaining the variation in abundance of plastic-degrading bacteria in cold-seep environments. Asterisks represent statistical significance: * indicates *P* < 0.05, ** indicates *P* < 0.01, and *** indicates *P* < 0.001. (**c**) Canonical correlation analysis revealing correlations between environmental geochemical parameters and the abundance of plastic-degrading bacteria.

After eight cycles of enrichment culture, 14 putative PET-degrading and 12 putative PP-degrading bacterial genera were identified across the 14 enrichment cultures. Among them, 13 genera exhibited clear niche partitioning in the natural cold-seep seawater and sediment communities (Fig. 7a). Overall, PET- and PP-degrading taxa were significantly more abundant in the seawater community than in the sediment community. The relative abundances of degraders in sediments ranged from 0.0019% to 2.11%, whereas the relative abundances of degraders in seawater ranged from 0.43% to 5.60%. In sediment communities, *Bacillus* and *Brevibacterium* were the predominant putative plastic degraders, while *Erythrobacter*, *Halomonas*, and *Paracoccus* dominated seawater communities. Because the PET and PP plastic plastispheres were incubated in a cold-seep seawater environment (Fig. 1a), the microorganisms recovered from the plastics and subsequent enrichment cultures were largely drawn from the seawater microbiome, which likely explained the higher abundances of PET- and PP-degrading bacteria detected in seawater communities compared with sediment communities. A previous study reported that the relative abundances of putative PE/PP-degrading bacteria in overlying river water and sediments of the Qinhuai River was below 0.25% (29). In contrast, the present study demonstrated that putative PET- and PP-degrading bacteria were widespread in cold-seep seawater, with a maximum relative abundance of up to 5.6%, suggesting that deep-sea cold seeps may act as a hotspot for plastic degradation.

Different environmental regional chemistries significantly influence the formation of local microbial communities. Therefore, Mantel tests and CCA analysis were applied to further reveal the key environmental drivers of plastic-degrading bacterial distribution in cold-seep sediments and seawater environments (Fig. 7b and c). The results showed that CH_4_, inorganic nitrogen (NO_3_^–^, NO_2_^–^, and NH_4_^+^), PO_4_^3–^, Ca^2+^, Mg^2+^, and SO_4_^2–^ significantly influenced plastic-degrading bacterial diversity in the cold seeps. With the exception of Mg^2+^, these variables were positively correlated with the abundance of putative plastic degraders, suggesting that more eutrophic cold seeps harbor greater abundances of plastic-degrading taxa. Methane is a key energy source in cold-seep ecosystems that drives biogeochemical processes that enrich hydrocarbon-degrading bacteria. Because petroleum-derived plastics are hydrocarbon polymers, these hydrocarbon degraders represent plausible candidates for plastic degradation. Accordingly, the results showed that environments with elevated ambient methane concentrations tended to support higher abundances of putative plastic-degrading bacteria (Fig. 7a; Table 2).

**TABLE 2:** Methane concentrations in seawater and sediment at different cold-seep sites

Sample	Methane (nmol/L)	Sample	Methane (nmol/g)
Seawater-1	117.72	Sediment-1	9,458.51
Seawater-2	91.02	Sediment-2	2,038.07
Seawater-3	59.47	Sediment-3	3,668.83
Seawater-4	51.28	Sediment-4	5,595.32
Seawater-5	124.07	Sediment-5	5,595.32

Environmental surveys revealed high abundances of putative PET- and PP-degrading microorganisms throughout the cold-seep sites. The physicochemical and nutrient conditions of cold seeps selectively favor metabolically versatile hydrocarbon-degrading taxa biochemically preadapted to attack polymeric hydrocarbons or their oxidation products (67, 68). The chemosynthetic food web typical of cold seeps further amplifies the prevalence of hydrocarbon-oxidizing and heterotrophic degraders. Many of these hydrocarbon-degrading bacteria encode monooxygenases, dioxygenases, and various esterases and hydrolases, which can act promiscuously or via co-metabolism on plastic polymer backbones or oxidatively depolymerized products (69). Consistent with this functional predisposition, higher relative abundances of putative plastic-degrading taxa were detected at sites with elevated methane concentrations. However, methane seepage may have a dual influence on the plastic-degrading potential of cold-seep plastispheres. On the one hand, methane-rich environments can enrich methane-oxidizing and hydrocarbon-degrading microorganisms, which may serve as potential sources of candidate plastic-degrading taxa because of their hydrocarbon-transforming metabolic capabilities. On the other hand, under *in situ* cold-seep conditions, methane is likely a more readily available carbon and energy source than recalcitrant PET or PP. These hydrocarbon-associated microorganisms may therefore preferentially utilize methane or methane-derived organic substrates rather than plastic polymers, thereby reducing the selective pressure for PET/PP degradation. Thus, although methane seepage may increase the reservoir of microorganisms with potential plastic-degrading capabilities, the actual *in situ* degradation efficiency of PET/PP may be limited and requires further assessment using cold-seep-simulated incubation experiments. Additionally, the abundances of plastic-degrading bacteria were positively correlated with inorganic nutrients. This was likely because the eutrophic cold-seep environment provided the resources and ecological conditions necessary to sustain higher abundances of plastic-degrading microorganisms. Higher nutrient contents enhanced the growth rate of heterotrophic microorganisms, stimulated extracellular enzyme production, and promoted the formation of dense biofilms and extracellular polymers on plastic surfaces. These factors contributed to the depolymerization of plastics and the uptake of released oligomers, leading to the enrichment of plastic-degrading bacteria.

### Conclusion

In this study, 1 year of *in situ* inoculation and 168 days of laboratory enrichment culture were combined to selectively enrich plastic-degrading bacteria from the cold-seep plastisphere. The results demonstrated that the enriched communities could utilize PET and PP plastics as carbon and energy sources for growth, with the cell abundances of cultures consistently increasing over time. SEM observations revealed grooves, cracks, and surface irregularities on PET and PP films after enrichment, indicating potential surface biodeterioration under laboratory conditions. During the cultivation process, the relative abundances of bacterial populations unable to utilize plastic films as carbon and energy sources gradually declined (e.g., *Kocuria*), while the abundances of potential plastic degraders progressively increased (e.g., *Halomonas*). Tracking ASV dynamics yielded a total of 24 PET-degrading and 22 PP-degrading candidate ASVs. These potential degraders exhibited an expanded spectrum of plastic degradation capabilities, and several taxa emerged as putative plastic degraders that were not previously reported as plastic-degrading taxa, including *Aeromicrobium*, *Salinisphaera*, and *Croceicoccus*. Environmental surveys demonstrated that the putative PET- and PP-degrading bacteria were widespread in natural cold-seep habitats. The results of correlation analysis suggest that eutrophic cold-seep sediment and seawater communities may possess greater potential for plastic degradation than less nutrient-rich environments.

The present study focused on the community-level enrichment and identification of candidate plastic-degrading taxa from the deep-sea cold-seep plastisphere. However, the isolation of pure cultures with confirmed PET- and PP-degrading capabilities remains to be accomplished, and their ecological relevance under *in situ* cold-seep conditions requires further verification. Future work should prioritize isolating and characterizing individual strains from these enrichment cultures, elucidating metabolic pathways of PET and PP transformation, and testing the degradation efficacy of different strains under cold seep-simulated conditions, including low temperature, high hydrostatic pressure, and methane-rich environments. Harnessing the adaptive traits of cold-seep microbial communities may provide valuable bioremediation strategies to mitigate plastic pollution in deep-sea ecosystems.

## Data Availability

All raw sequences of 16S rRNA gene amplicon sequencing were deposited in the National Center for Biotechnology Information (NCBI) under PRJNA1366089. In addition, the processed ASV tables, representative ASV sequences, sample metadata, environmental parameter table, and analysis scripts used in this study are available in a public GitHub repository: https://github.com/YongjiHuang/Cold-seep-plastisphere-PET-PP-enrichment.git.

## SUPPLEMENTAL MATERIAL

Supplemental material spectrum.01632-26-s0001.docx Tables S1 and S2; Fig. S1 and S2.

ASM does not own the copyrights to Supplemental Material that may be linked to, or accessed through, an article. The authors have granted ASM a non-exclusive, world-wide license to publish the Supplemental Material files. Please contact the corresponding author directly for reuse.
