# Supplementary material for: Diversity and distribution of potential polyethylene terephthalate and polypropylene plastic-degrading bacteria communities in deep-sea cold-seep ecosystems
Source: Microbiol Spectr. 2026 Oct 7;14(11):e01632-26. doi: 10.1128/spectrum.01632-26 (PMC13729202; doi:10.1128/spectrum.01632-26)
Supplement: Supplementary file 1 [file spectrum.01632-26-s0001.docx]

**Supplementary materials for**

**Diversity and distribution of potential polyethylene terephthalate and** **polypropylene plastic-degrading bacteria communities in deep-sea cold seep ecosystems**

Yongji Huang^a^, Jingchun Feng^a,b,^^[1]^*, Qixuan Wu^b^, Yingli Zhou^a^, Si Zhang^a,b,c,*^

^a^ Guangdong Basic Research Center of Excellence for Ecological Security and Green Development, Southern Marine Science and Engineering Guangdong Laboratory (Guangzhou), Guangzhou, 511458, China

^b^ Guangdong Basic Research Center of Excellence for Ecological Security and Green Development, School of Ecology, Environment and Resources, Guangdong University of Technology, Guangzhou 510006, China

^c^ South China Sea Institute of Oceanology, Chinese Academy of Sciences, Guangzhou 510301, China

**Tables and Figures**

Table S1 Composition of modified inorganic salt medium.

| Ingredient | Concentration (g/L) |
| --- | --- |
| K_2_HPO_4_ | 0.7 |
| KH_2_PO_4_ | 0.7 |
| MgSO_4_·7H_2_O | 0.7 |
| NH_4_NO_3_ | 1 |
| NaCl | 28 |
| CaCl_2_·2H_2_O | 0.02 |
| FeSO_4_·7H_2_O | 0.002 |
| ZnSO_4_·7H_2_O | 0.002 |
| MnSO_4_·H_2_O | 0.001 |

Table S2 Summary of isolated pure culture strains with plastic-degrading potential.

| Isolated strains | Enrichment source | Degradation type |
| --- | --- | --- |
| *Dietzia maris* | ROV1-PET-Y-1 | PET |
| *Bacillus altitudinis* | ROV2-PP-P-2 | PP |
| *Brevibacterium casei* | ROV2-PET-Y-1 | PET |
| *Cytobacillus firmus* | ROV2-PET-Y-3 | PET |
| *Cytobacillus oceanisediminis* | ROV1-PET-Y-2 | PET |
| *Dietzia maris* | ROV1-PP-Y-1 | PP |
| *Halomonas sp.* | ROV2-PP-Y-1 | PP |
| *Kocuria sp.* | ROV2-PET-Y-3 | PET |
| *Salinisphaera shabanensis* | ROV1-PET-Y-3 | PET |
| *Vreelandella titanicae* | ROV2-PP-Y-1 | PP |

Fig. S1 Good's coverage index for enriched microbial communities.

Fig. S2 α-diversity of enriched microbial communities in different culture cycles including, the (a) observed amplicon sequence variant (ASV), (b) Chao1 richness, (c) phylogenetic diversity, (d) and Shannon diversity indices.

1. *Corresponding author. E-mail address: fengjc@gdut.edu.cn. (J. C. Feng), zhisimd@scsio.ac.cn (S. Zhang)

   Tel: +86-20-39322141. Fax: 020-39322141
